# Multicomponent synthesis of pyrimido[4,5-*b*] quinolines over a carbocationic catalytic system

**DOI:** 10.1038/s41598-023-43793-5

**Published:** 2023-10-01

**Authors:** Ahmad Reza Moosavi-Zare, Raha Najafi

**Affiliations:** Department of Chemical Engineering, Hamedan University of Technology, Hamedan, 65155 Iran

**Keywords:** Organic chemistry, Catalysis, Organocatalysis

## Abstract

Trityl chloride (TrCl) was efficiently used as a neutral catalyst for the multicomponent cyclization reaction of an aldehyde with dimedone and 6-amino-1,3-dimethyluracil for the preparation of pyrimido[4,5-*b*] quinolines in chloroform under reflux condition.

## Introduction

Organic catalysts with high stability, insensitivity to moisture and oxygen, specific molecular structure, low toxicity and the absence of metal in their structure are notable for the catalyze of the organic reactions^1,2^.

Recently, the application of trityl chloride as an organic catalyst has been considered in organic synthesis due to some advantages such as cheapness and commercially available. Also, neutral and mild reaction condition in terms of temperature and pressure is another benefits for trityl chloride as a catalyst^3^.

Trityl chloride was applied as an organic catalyst for the preparation of various organic compounds such as bis(indolyl) methanes^3^, *N*-sulfonyl imines^4^, 1-amidoalkyl-2-naphtols^5^, 12-aryl-8,9,10,12-tetrahydrobenzo[a]-xanthen-11-ones^6^, 1-thioamido-alkyl-2-naphthols, 1-carbamato-alkyl-2-naphthols^7^, 3-(2,6-diarylpyridin-4-yl)-1H-indoles, 2,4,6-triarylpyridines^8^, pyranopyrazoles^9^, gem-bisamides^10^, 1,2,4,5-tetrasubstitutedimidazoles^11^, and α,α′-bis(arylidene)cycloalkanones^12^.

Pyrimido[4,5-*b*]quinolones are important compounds in medicinal chemistry due to some biological properties such as antifungal^13^, antimalarial^14^, anticancer^15,16^, antiviral^17^, antihistaminic^18^, anti-oxidant, anti-microbial^19^, and anti-inflammatory activities^19,20^. The one pot multi-component synthesis of pyrimido[4,5-*b*]quinolones by the reaction of aldehyde with dimedone and 6-amino-1,3-dimethyluracil is an important method for the preparation of these compounds^19^. Multi-component reactions are notable for the chemists due to prepare of the target product in one step without the produce of side products. High atomic economy, high yields, short reaction times, saving energy, reaction times, materials and solvents and compliance with green chemistry protocols are some important advantages of these reactions^21–26^.

Various catalysts were used in the multi-component synthesis of pyrimido[4,5-*b*]quinolines such as [TSSECM]^27^, SBA-15/PrN(CH_2_PO_3_H_2_)_2_^28^, Nano-[Fe_3_O_4_@SiO_2_/N-propyl-1-(thiophen-2-yl)ethanimine][ZnCl_2_]^29^, [H_2_-DABCO][ClO_4_]_2_^30^, nano-[Fe_3_O_4_@- SiO_2_@R-NHMe_2_][H_2_PO_4_]^31^, *N,N*-diethyl-*N*-sulfoethanaminium chloride^32^, Fe_3_O_4_@Cellulose sulfuric acid^33^, [bmim]Br^34^, nanocrystalline MgO^35^, glycolic acid-supported cobalt ferrite^36^, Agar-entrapped sulfonated DABCO^37^, [C_4_(DABCO)_2_]·2OH^38^, DABCO^39^, Nano‑[Cu‑4C3NSP](Cl)_2_^40^, and Cellulose sulfuric acid^41^, nano-[Fe_3_O_4_@SiO_2_@BDIL]^42^ and Cs_2.3_H_0.7_PW_10_Mo_2_O_40_^43^. Most of the reported methods are performed by acidic or basic catalysts. In the presented work, the preparation of pyrimido[4,5-b]quinolines was reported in the presence of trityl chloride (TrCl) as a neutral and cheap compound which is commercially available. This method was carried out under mild reaction condition. In the presented work, it is interesting that, the reaction was carried out using carbocationic catalytic system.

**Figure 1:**
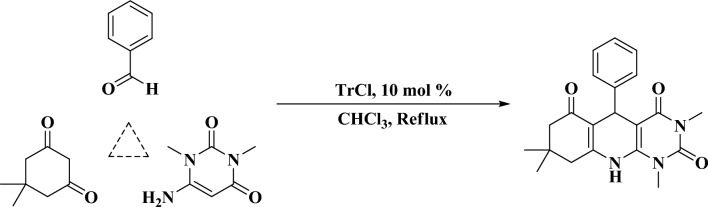
The preparation of pyrimido[4,5-*b*]quinolines.

In continues to our investigation on the catalytic application of carbocationic system to carry out the organic reactions, we have used of trityl chloride (TrCl) as a neutral catalyst for the preparation of pyrimido[4,5-*b*] quinolines by the multi component reaction of aromatic aldehydes with dimedone and 6-amino-1,3-dimethyluracil (Fig. 1).

## Results and discussion

In the first step, to find the best reaction condition, the reaction of 2, 4-dichlorobenzaldehyde with dimedone and 6-amino-1,3-dimethyluracil was selected as a model reaction and kinds of catalysts, catalyst amounts and temperature were studied on this reaction. Also, the model reaction was carried out in various solvents such as chloroform, ethanol, ethyl acetate, n-hexane, acetone, dichloromethane and acetonitrile in comparison with solvent-free condition. According that, the best result was obtained using 10 mol % of trityl chloride as a catalyst in chloroform under reflux condition (Table 1).

**Table 1:** Effect of catalyst amount, temperature and solvent on the reaction between 2, 4-dichlorobenzaldehyde (1 mmol) with dimedone (1 mmol) and 6-amino-1,3-dimethyluracil (1 mmol).

Entry	Catalyst (Mol%)	Solvent	Temp. (°C)	Time (min)	Yield^a^ (%)
1	TrCl (10)	CHCl_3_	Reflux	130	92
2	TrCl (5)	CHCl_3_	Reflux	130	68
3	TrCl (15)	CHCl_3_	Reflux	130	90
4	TrCl (10)	CHCl_3_	r.t	130	52
5	TrCl (10)	Solvent free	60 °C	130	Trace
6	-	CHCl_3_	Reflux	130	Trace
7	MMTrCl (10)	CHCl_3_	Reflux	130	75
8	DMTrCl (10)	CHCl_3_	Reflux	130	53
9	TrCl (10)	Ethanol	Reflux	130	48
10	TrCl (10)	Ethyl acetate	Reflux	130	59
11	TrCl (10)	n-Hexane	Reflux	130	44
12	TrCl (10)	Acetone	Reflux	130	41
13	TrCl (10)	CH_2_Cl_2_	Reflux	130	45
14	TrCl (10)	Acetonitrile	Reflux	130	23

^a^Isolated yield.

**Figure 2:**
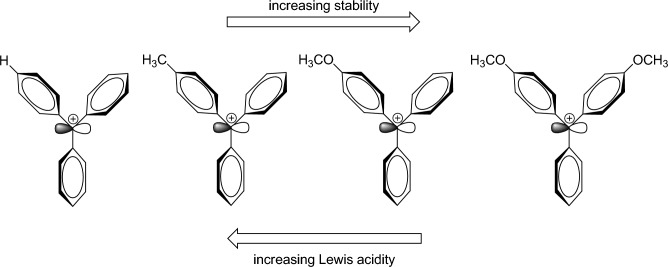
The comparison of trityl carbocation derivatives with each other in term of acidity and stability.

The catalytic effect of trityl chloride has been compared with its other derivatives such as trityl alcohol, monomethoxytrityl chloride and dimethoxytrityl chloride on the three-component reaction of dimedone, 6-amino-1,3-dimethyluracil and 2, 4-dichlorobenzaldehyde. In this study, trityl chloride showed the best catalytic performance and in the presence of this catalyst, the shortest reaction time and the highest efficiency for the desired product were obtained. The presence of electron-donating groups on the trityl chloride ring stabilizes the carbocation and reduces its acid strength. The order of stability and acid strength of some carbocations of the trityl derivatives is specified in Fig. 2^44^.

**Figure 3:**
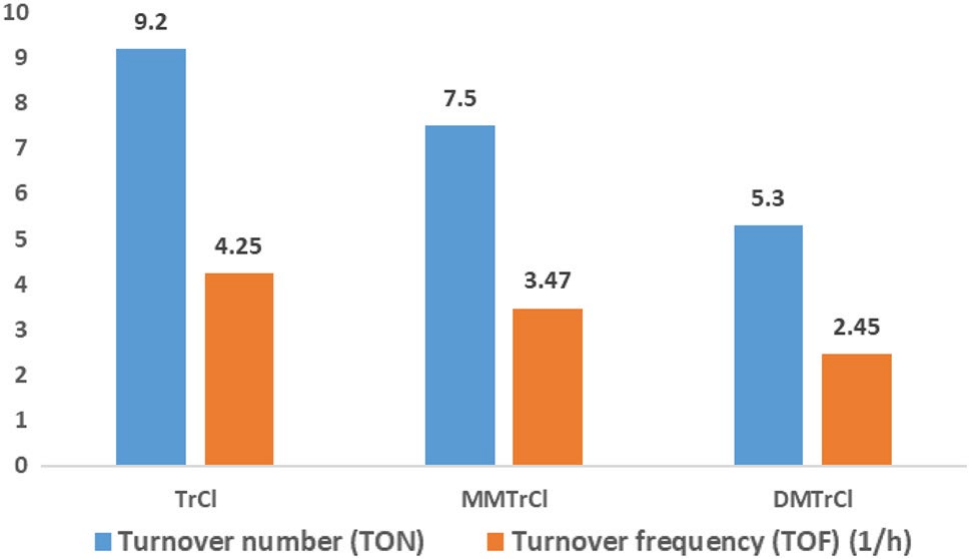
Catalytic activity of various triarylmethyl chlorides on the reaction of 2, 4-dichlorobenzaldehyde with dimedone and 6-amino-1,3-dimethyluracil in chloroform under reflux condition.

Turn-over frequency (TOF) and Turn-over number (TON) of TrCl in comparison with other derivatives of TrCl such as monomethoxytrityl chloride and dimethoxytrityl chloride on the three-component reaction of dimedone, 6-amino-1,3-dimethyluracil and 2, 4-dichlorobenzaldehyde to show the superiority of TrCl as a catalyst for the synthesis of pyrimido[4,5-*b*]quinolines were given in Fig. 3. As it is shown in Fig. 3, indicates that TrCl is more efficient than the other catalysts in terms of TOF and TON for the preparation of this compounds.

After the optimization of the reaction condition, various aromatic aldehydes containing releasing or withdrawing groups and halogen on their ring, were reacted with dimedone and 6-amino-1,3-dimethyluracil to give pyrimido[4,5-*b*]quinolines derivatives. All products were obtained in high yields and short reaction times (Table 2).

**Table 2:** The preparation of pyrimido[4,5-*b*]quinolones in the presence of TrCl.

Entry	Product	Time (min)	Yield^a^ (%)	M.p. °C (Lit.)^ref^
1		130	79	253–255 (266–269)^30^
2		130	84	350–355 (> 300)^27^
3		130	92	340 (> 300)^32^
4		130	88	279–280 (284–288)^20^
5		130	77	332–335 (> 300)^28^
6		130	73	296–299 (> 300)^37^
7		130	75	307–309 (304–306)^31^
8		130	78	273–276 (278–280)^41^
9		130	76	303–305
10		130	88	290–293 (296–297)^42^
11		130	74	298–300
12		130	83	300–303 (> 300)^43^
13		130	85	291–295 (293–295)^40^

^a^The isolated yield.

**Figure 4:**
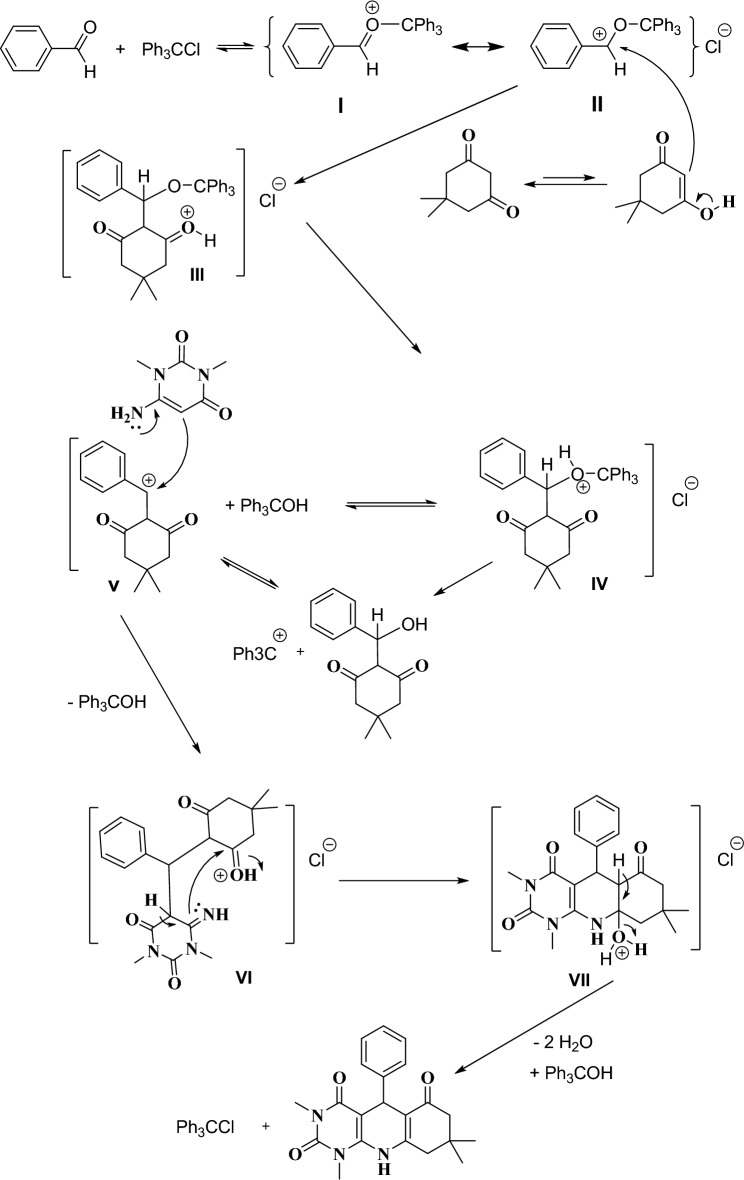
The proposed mechanism for the preparation of pyrimido[4,5-*b*]quinolines.

To discuss the mechanism for the preparation of the pyrimido[4,5-*b*]quinolines compounds in the presence of carbocationic catalytic system under neutral media, as it is reported on previous literature^3–12^, firstly, aldehyde was converted to activated forms (**I** and **II**) by the reaction with trityl cation which in situ generated from trityl chloride. Then, dimedone in enole form reacted with activated forms of aldehyde (**I** and **II**) to give (**III**) which could be converted to (**IV**) and (**V**). Then, 6-amino-1,3-dimethyluracil was reacted with intermediate (**V**) to prepare (**VI**). Lastly, by the intramolecular nucleophilic attack in intermediate (**VI**), the expected product was synthesized after removing of one molecule of H_2_O. Trityl chloride was regenerated by the reaction of trytil alcohol with (**VII**) and removing of one molecule of H_2_O (Fig. 4).

## Materials and methods

All chemicals were purchased from Merck or Fluka Chemical Companies. The known products were identified by comparison of their melting points and spectral data with those reported in the literature. Progress of the reactions was monitored by TLC using silica gel SIL G/UV 254 plates. The ^1^H NMR (250 MHz) and ^13^C NMR (62.5 MHz) were recorded on a Bruker Avance DPX FT-NMR spectrometer (δ in ppm). Melting points were recorded on a Büchi B-545 apparatus in open capillary tubes.

### General procedure for the synthesis of pyrimido[4,5-b]quinolines

In a round-bottomed flask which connected to a reflux condenser, aromatic aldehydes (1 mmol), dimedone (1 mmol, 0.140 g), 6-amino-1,3-dimethyluracil (1 mmol, 0.155 g) and trityl chloride (TrCl) (10 mol %, 0.0278 g) as a catalyst were added and stirred in chloroform (10 mL) as a solvent under reflux condition for appropriate time (Table 2). After completion of the reaction as monitored by TLC, the solvent was removed and finally, the desired product was purified by the recrystallization in aqueous ethanol (70%).

### Selected spectral data of compounds

#### 5-(2-chlorophenyl)-1,3,8,8-tetramethyl-7,8,9,10-tetrahydropyrimido[4,5-b]quinoline-2,4,6(1H,3H,5H)-trione

White Solid; M.p: 350–355 °C;IR (KBr, cm^−1^): 3281, 3224, 3094, 2960, 1702, 1641, 753, 538; ^1^H NMR (250 MHz, DMSO-*d*_*6*_): *δ* 0.85 (s, 3H), 1.00 (s, 3H), 1.93 (d, *J* = 16 Hz, 1H), 2.16 (d, *J* = 16 Hz, 1H), 2.52 (s, 2H), 3.01 (s, 3H), 3.42 (s, 3H), 5.15 (s, 1H), 7.05 (d,* J* = 6.75 Hz, 1H), 7.14 (d, *J* = 7.25 Hz, 2H), 7.29 (s, 1H), 8.97 (s, 1H); ^13^C-NMR (DMSO-*d*_*6*_, 62.5 MHz): δ 26.7, 27.9, 29.5, 30.6, 32.3, 34.1, 50.5, 89.9, 111.2, 126.7, 127.7, 129.5, 132.4, 133.0, 143.8, 144.5, 150.2, 150.9, 160.8, 194.7.

#### 5-(2,4-dichlorophenyl)-1,3,8,8-tetramethyl-5,8,9,10-tetrahydropyrimido[4,5-b]quinoline-2,4,6(1H,3H,7H)-trione

White Solid; M.p: 340 °C;IR (KBr, cm^−1^): 3278, 3218, 3093, 2950, 2875, 1705, 1661, 1643, 1610, 1495,1379, 1211, 1045,755, 508; ^1^H NMR (250 MHz, DMSO-*d*_*6*_): δ 0.85 (s, 3H), 1.00 (s, 3H), 1.94 (d,* J* = 16.25 Hz, 1H), 2.16 (d, *J* = 16 Hz, 1H), 2.49 (d, *J* = 12 Hz, 2H), 3.01(s, 3H), 3.41 (s, 3H), 5.10 (s, 1H), 7.22 (d, *J* = 8 Hz, 2H), 7.30 (s, 1H), 8.98 (s, 1H); ^13^C-NMR (DMSO-*d*_*6*_, 62.5 MHz): δ 26.8, 27.9, 29.4, 30.6, 32.3, 34.0, 49.8, 50.4, 89.5, 110.8, 126.,9 128.7, 131.2, 133.6, 134.0, 143.0, 144.6,150.5,150.9,160.9,194.8.

#### 1,3,8,8-tetramethyl-5-(p-tolyl)-5,8,9,10-tetrahydropyrimido[4,5-b]quinoline-2,4,6(1H,3H,7H)-trione

White Solid; M.p: 332–335 °C;IR (KBr, cm^−1^): 3281, 3220, 3090, 2961, 1702, 1662, 1603, 1507, 1380, 962, 756; ^1^H NMR (250 MHz, DMSO-*d*_6_): δ 0.86 (s, 3H), 1.01 (s, 3H), 1.99 (d, *J* = 16 Hz, 1H), 2.16 (s, 3H), 2.18 (d, *J* = 16 Hz, 1H), 2.50 (d, *J* = 12.5 Hz, 2H), 3.05 (s, 3H), 3.42 (s, 3H), 4.80 (s, 1H), 6.94 (d, *J* = 7.5 Hz, 1H), 7.07 (d, *J* = 7.5 Hz, 2H), 8.94 (s, 1H); ^13^C-NMR (DMSO-*d*_6_, 62.5 MHz): δ 21.0, 26.9, 28.0, 29.5, 30.5, 32.5, 33.7, 50.5, 90.7, 112.2, 127.9, 128.6, 135.2, 143.9, 144.1, 149.7, 150.9, 161.1, 194.9.

#### 5-(4-methoxyphenyl)-1,3,8,8-tetramethyl-5,8,9,10-tetrahydropyrimido[4,5-b]quinoline-2,4,6(1H,3H,7H)-trione

White Solid; M.p: 307–309 °C; ^1^H NMR (250 MHz, DMSO-*d*_6_): δ 0.86 (s, 3H), 1.01 (s, 3H), 2.00 (d, *J* = 16.00 Hz, 1H), 2.18 (d, *J* = 16.00 Hz, 1H), 2.48–2.54 (m, 2H), 3.06 (s, 3H), 3.41 (s, 3H), 3.64 (s, 3H), 4.78 (s, 1H), 6.70 (d, *J* = 8.00 Hz, 1H), 7.09 (d, *J* = 7.75 Hz, 1H), 8.94(s, 1H); ^13^C-NMR (DMSO-*d*_6_, 62.5 MHz): δ 26.9, 28.0, 29.5, 30.6, 32.5, 33.3, 44.7, 50.5, 55.3, 90.8, 112.3, 113.5, 125.2, 129.0, 139.1, 144.0, 149.6, 151.0, 157.8, 161.1, 195.0, 202.2.

#### 5-(2-hydroxy-3-methoxyphenyl)-1,3,8,8-tetramethyl-5,8,9,10-tetrahydropyrimido[4,5-b]quinoline-2,4,6(1H,3H,7H)-trione

White Solid; M.p: 298–300 °C; ^1^H NMR (250 MHz, DMSO-*d*_6_): δ 0.91 (s, 3H), 1.02 (s, 3H), 2.04 (d, *J* = 15.75 Hz, 1H), 2.23 (d, *J* = 15.75 Hz, 1H), 2.57 (d, *J* = 8.75 Hz, 2H), 3.08 (s, 3H), 3.67 (s, 3H), 4.96 (s, 1H), 6.55–6.64 (m, 3H), 9.09 (s, 1H), 9.15(s, 1H); ^13^C-NMR (DMSO-*d*_6_, 62.5 MHz): δ 27.0, 28.3, 29.4, 30.8, 32.5, 50.3, 55.9, 110.5, 119.6, 120.9, 134.3, 151.4, 196.0.

## Conclusions

In summary, TrCl was used as a homogeneous organocatalyst for the synthesis of pyrimido[4,5-*b*]quinolines derivatives in neutral condition. The products were prepared in high yields and short reaction times. The low cost of trityl choride, commercially availability of the catalyst, short reaction times and the avoidance of harsh acidic conditions are important advantages of this work.

### Supplementary Information

Supplementary Information.

## Data Availability

All data generated or analysed during this study are included in this published article (and its Supplementary Information files).

## References

[1] Pandey J, Anand N, Tripathi RP (2009). L-Proline catalyzed multicomponent reaction of 3,4-dihydro-(2H)-pyran, urea/thiourea, and aldehydes: Diastereoselective synthesis of hexahydropyrano pyrimidinones (thiones). Tetrahedron.

[2] Chandrasekhar S, Johny K, Reddy CR (2009). Proline–threonine dipeptide as an organocatalyst for the direct asymmetric aldol reaction. Tetrahedron Asymmetry.

[3] Khalafi-Nezhad A, Parhami A, Zare A, Moosavi Zare AR, Hasaninejad A, Panahi F (2008). Trityl chloride as a novel and efficient organic catalyst for room temperature preparation of bis (indolyl) methanes under solvent-free conditions in neutral media. Synthesis.

[4] Khalafi-Nezhad A, Parhami A, Zare A, Nasrollahi Shirazi A, Moosavi-Zare AR, Hasaninejad A (2008). Triarylmethyl chlorides as novel, efficient, and mild organic catalysts for the synthesis of N-sulfonyl imines under neutral conditions. Can. J. Chem..

[5] Khazaei A, Zolfigol MA, Moosavi-Zare AR, Zare A, Parhami A, Khalafi-Nezhad A (2010). Trityl chloride as an efficient organic catalyst for the synthesis of 1-amidoalkyl-2-naphtols in neutral media at room temperature. Appl. Catal. A Gen..

[6] Khazaei A, Zolfigol MA, Moosavi-Zare AR, Zare A, Khojasteh M, Asgari Z, Khakyzadeh V, Khalafi-Nezhad A (2012). Organocatalyst trityl chloride efficiently promoted the solvent-free synthesis of 12-aryl-8,9,10,12-tetrahydrobenzo[a]-xanthen-11-ones by in situ formation of carbocationic system in neutral media. Catal. Commun..

[7] Khazaei A, Zolfigol MA, Moosavi-Zare AR, Abi F, Zare A, Kaveh H, Khakyzadeh V, Kazem-Rostami M, Parhami A, Torabi-Monfared H (2013). Discovery of an in situ carbocationic system using trityl chloride as a homogeneous organocatalyst for the solvent-free condensation of β-naphthol with aldehydes and amides/thioamides/alkyl carbamates in neutral media. Tetrahedron.

[8] Moosavi-Zare AR, Zolfigol MA, Rezanejad Z (2016). Trityl chloride promoted the synthesis of 3-(2,6-diarylpyridin-4-yl)-1H-indoles and 2,4,6-triarylpyridines by in situ generation of trityl carbocation and anomeric based oxidation in neutral media. Can. J. Chem..

[9] Moosavi-Zare AR, Zolfigol MA, Mousavi-Tashar A (2016). Synthesis of pyranopyrazole derivatives by in situ generation of trityl carbocation under mild and neutral media. Res. Chem. Intermed..

[10] Moosavi-Zare AR, Goudarziafshar H, Jalilian Z, Hajilouie Z (2022). The synthesis of gem-bisamides using a carbocationic catalytic system in neutral media. Org. Prep. Proced. Int..

[11] Moosavi-Zare AR, Asgari Z, Zare A, Zolfigol MA, Shekouhy M (2014). One pot synthesis of 1, 2, 4, 5-tetrasubstituted-imidazoles catalyzed by trityl chloride in neutral media. RSC Adv..

[12] Zare A, Merajoddin M, Hasaninejad A, Moosavi-Zare AR, Khakyzadeh V (2013). Study of in situ generation of carbocationic system from trityl chloride (Ph3CCl) which efficiently catalyzed cross-aldol condensation reaction. C. R. Chimie.

[13] Drabczynska A, Muller CE, Schiedel A, Schumacher B, Wojciechowska JK, Fruzinski A, Zobnina W, Yuzlenko O, Kiec-Kononowicz K (2007). Phenylethyl-substituted pyrimido[2,1-f]purinediones and related compounds: Structure–activity relationships as adenosine A1 and A2A receptor ligands. Bioorg. Med. Chem..

[14] Joshi AA, Viswanathan CL (2006). Recent developments in antimalarial drug discovery. Agents Med. Chem..

[15] Valderrama JA, Colonelli P, Vasquez D, Gonzalez MF, Rodríguez JA, Theoduloz C (2008). Studies on quinones. Part 44: Novel angucyclinone N-heterocyclic analogues endowed with antitumoral activity. Bioorg. Med. Chem..

[16] Alqasoumi SI, Al-Taweel AM, Alafeefy AM, Noaman E, Ghorab MM (2010). Novel quinolines and pyrimido [4, 5-*b*] quinolines bearing biologically active sulfonamide moiety as a new class of antitumor agents. Eur. J. Med. Chem..

[17] Nair V, Chi G, Shu Q, Julander J, Smee DF (2009). A heterocyclic molecule with significant activity against dengue virus. Bioorg. Med. Chem. Lett..

[18] Alagarsamy V (2004). Synthesis and pharmacological investigation of some novel 2-methyl-3-(substituted methylamino)-(3H)-quinazolin-4-ones as histamine H1-receptor blockers. Pharmazie.

[19] El-Gazzar ABA, Hafez HN, Nawwar GAM (2009). New acyclic nucleosides analogues as potential analgesic, anti-inflammatory, anti-oxidant and anti-microbial derived from pyrimido [4, 5-*b*] quinolones. Eur. J. Med. Chem..

[20] Alagarsamy V, Venkatesaperumal R, Vijayakumar S, Angayarkanni T, Pounammal P, Senthilganesh S, Kandeeban S (2002). Synthesis and pharmacological investigation of some novel 2-phenyl-3-(substituted methyl amino) quinazolin-4 (3H)-ones as H1-receptor blockers. Pharmazie.

[21] Baghernejad B, Harzevili MR (2021). Nano-cerium oxide/aluminum oxide: An efficient and useful catalyst for the synthesis of tetrahydro[a]xanthenes-11-one derivatives. Chem. Methodol..

[22] Moosavi-Zare AR, Goudarziafshar H, Jalilian Z, Hosseinabadi F (2022). Efficient pseudo-six-component synthesis of tetrahydro-pyrazolopyridines using [Zn-2BSMP]Cl_2_. Chem. Methodol..

[23] Abedini E, Shaterian HR (2023). L-Leucine supported on silica gel encapsulating γ-Fe_2_O_3_ nanoparticles: A new recoverable magnetic catalyst for preparation of 1, 3-thiazole derivatives. Eurasian Chem. Commun..

[24] Baghernejad B, Taromsari SMH (2022). Aqueous media preparation of 2-amino-4H-benzopyran derivatives using cerium oxide nanoparticles as a recyclable catalyst. Asian J. Green Chem..

[25] Muhiebes RM, Fatolahi L, Sajjadifar S (2023). L-proline catalyzed multicomponent reaction for simple and efficient synthesis of tetrahydropyridines derivatives. Asian J. Green Chem..

[26] Deb ML, Borpatra PJ, Baruah PK (2019). A one-pot catalyst/external oxidant/solvent-free cascade approach to pyrimidines via a 1,5-hydride transfer. Green Chem..

[27] Zare A, Dianat M, Eskandari MM (2020). A novel organic–inorganic hybrid material: production, characterization and catalytic performance for the reaction of arylaldehydes, dimedone and 6-amino-1,3-dimethyluracil. New J. Chem..

[28] Jalili F, Zarei M, Zolfigol MA, Rostamnia S, Moosavi-Zare AR (2020). SBA-15/PrN(CH_2_PO_3_H_2_)_2_ as a novel and efficient mesoporous solid acid catalyst with phosphorous acid tags and its application on the synthesis of new pyrimido[4,5-b]quinolones and pyrido[2,3-*d*]pyrimidines via anomeric based oxidation. Microporous Mesoporous Mater..

[29] Esmaili S, Moosavi-Zare AR, Khazaei A (2022). Nano-[Fe3O4@SiO2/N-propyl-1-(thiophen-2-yl)ethanimine][ZnCl2] as a nano magnetite Schiff base complex and heterogeneous catalyst for the synthesis of pyrimido[4,5-*b*]quinolones. RSC Adv..

[30] Shirini F, Langarudi MSN, Daneshvar N, Jamasbi N, Irankhah-Khanghah M (2018). Preparation and characterization of [H2-DABCO][ClO_4_]_2_ as a new member of DABCO-based ionic liquids for the synthesis of pyrimido[4,5-*b*]-quinoline and pyrimido[4,5-*d*]pyrimidine derivatives. J. Mol. Struct..

[31] Zare A, Lotfifar N, Dianat M (2020). Preparation, characterization and application of nano-[Fe_3_O_4_@-SiO_2_@R-NHMe_2_][H_2_PO_4_] as a novel magnetically recoverable catalyst for the synthesis of pyrimido[4,5-*b*]quinolones. J. Mol. Struct..

[32] Zare A, Dianat M (2021). A highly efficient and green approach for the synthesis of pyrimido[4,5-*b*]quinolines using N, N-diethyl-N-sulfoethanaminium chloride. Z. Naturforsch..

[33] Osanlou F, Nemati F, Sabaqian S (2017). An eco-friendly and magnetized biopolymer cellulose-based heterogeneous acid catalyst for facile synthesis of functionalized pyrimido[4,5-*b*]quinolines and indeno fused pyrido[2,3-*d*]pyrimidines in water. Res. Chem. Intermed..

[34] Shi D-Q, Ni S-N, Yang F, Shi J-W, Dou G-L, Li X-Y, Wang X-S, Ji S-J (2008). An Efficient synthesis of pyrimido[4,5-*b*]quinoline and indeno[2′,1′:5,6]pyrido[2,3-d]pyrimidine derivatives via multicomponent reactions in ionic liquid. J. Heterocyclic Chem..

[35] Rad AM, Mokhtary M (2015). Efficient one-pot synthesis of pyrido[2,3-d]pyrimidines catalyzed by nanocrystalline MgO in water. Int. Nano Lett..

[36] Gholami A, Mokhtary M, Nikpassand M (2020). Glycolic acid-supported cobalt ferrite-catalyzed one-pot synthesis of pyrimido[4,5-b]quinoline and indenopyrido[2,3-d]pyrimidine derivatives. Appl. Organomet Chem..

[37] Moghaddampour IM, Shirini F, Langarudi MSN (2021). Agar-entrapped sulfonated DABCO: Agelly acidic catalyst for the acceleration of one-pot synthesis of 1,2,4-triazoloquinazolinone and some pyrimidine derivatives. J. Mol. Struct..

[38] Jolodar OG, Shirini F, Seddighi M (2017). Efficient synthesis of pyrano[2,3-*d*]pyrimidinone and pyrido[2,3-d]pyrimidine derivatives in presence of novel basic ionic liquid catalyst. Chin. J. Catal..

[39] Esmaili S, Moosavi-Zare AR, Khazaei A, Najafi Z (2022). The synthesis of novel pyrimido[4,5-*b*] quinolines containing benzyloxy and 1,2,3-triazole moieties by DABCO as a basic catalys. ACS Omega.

[40] Moosavi-Zare AR, Goudarziafshar H, Bahrami Z (2023). Nano-[Cu-4C3NSP](Cl)_2_ as a new catalyst for the preparation of pyrimido[4,5-*b*]quinoline derivatives. Res. Chem. Intermed..

[41] Azimi SC (2014). Cellulose sulfuric acid catalyzed multicomponent reaction for efficient synthesis of pyrimido and pyrazolo[4,5-*b*]quinolines under solvent-free conditions. Iran. J. Catal..

[42] Zare A, Barzegar M (2020). Dicationic ionic liquid grafted with silica-coated nano-Fe_3_O_4_ as a novel and efficient catalyst for the preparation of uracil-containing heterocycles. Res. Chem. Intermed..

[43] Khillare KR, Aher DS, Chavan LD, Shankarwar SG (2021). Cesium salt of 2-molybdo-10-tungstophosphoric acid as an efficient and reusable catalyst for the synthesis of uracil derivatives via a green route. RSC Adv..

[44] Naidu VR, Ni S, Franzen J (2015). The carbocation: A forgotten Lewis acid catalyst. ChemCatChem.

