# Supplementary material for: Multicomponent synthesis of pyrimido[4,5-b] quinolines over a carbocationic catalytic system
Source: Sci Rep. 2023 Oct 1;13:16501. doi: 10.1038/s41598-023-43793-5 (PMC10543543; doi:10.1038/s41598-023-43793-5)

**Supporting Information**

**Multicomponent synthesis of pyrimido[4,5-b] quinolines over a carbocationic catalytic system**

Ahmad Reza Moosavi-Zare,* Raha Najafi

Department of Chemical Engineering, Hamedan University of Technology, Hamedan, 65155, Iran.

**Spectral data of compounds:**

**5-(2-chlorophenyl)-1,3,8,8-tetramethyl-7,8,9,10-tetrahydropyrimido[4,5-b]quinoline-2,4,6(1H,3H,5H)-trione**

White Solid; M.p: 350-355 °C;IR (KBr, cm^-1^): 3281, 3224, 3094, 2960, 1702, 1641, 753, 538; ^1^H NMR (250 MHz, DMSO-*d_6_*): *δ* 0.85 (s, 3H), 1.00 (s, 3H), 1.93 (d, *J* = 16 Hz, 1H), 2.16 (d, *J* = 16 Hz, 1H), 2.52 (s, 2H), 3.01 (s, 3H), 3.42 (s, 3H), 5.15 (s, 1H), 7.05 (d, *J* = 6.75 Hz, 1H), 7.14 (d, *J* = 7.25 Hz, 2H), 7.29 (s, 1H), 8.97 (s, 1H); ^13^C-NMR (DMSO-*d_6_*, 62.5 MHz): δ 26.7, 27.9,29.5, 30.6, 32.3, 34.1, 50.5, 89.9, 111.2, 126.7, 127.7, 129.5, 132.4, 133.0, 143.8, 144.5, 150.2, 150.9, 160.8, 194.7.

**5-(2,4-dichlorophenyl)-1,3,8,8-tetramethyl-5,8,9,10-tetrahydropyrimido[4,5-b]quinoline-2,4,6(1H,3H,7H)-trione**

White Solid; M.p: 340 °C;IR (KBr, cm^-1^): 3278, 3218, 3093, 2950, 2875, 1705, 1661, 1643, 1610, 1495,1379, 1211, 1045,755, 508; ^1^H NMR (250 MHz, DMSO-*d_6_*): δ 0.85 (s, 3H), 1.00 (s, 3H), 1.94 (d, *J* = 16.25 Hz, 1H), 2.16 (d, *J* = 16 Hz, 1H), 2.49 (d, *J* = 12 Hz, 2H), 3.01(s, 3H), 3.41 (s, 3H), 5.10 (s, 1H), 7.22 (d, *J* = 8 Hz, 2H), 7.30 (s, 1H), 8.98 (s, 1H); ^13^C-NMR (DMSO-*d_6_*, 62.5 MHz): δ 26.8, 27.9, 29.4, 30.6, 32.3, 34.0, 49.8, 50.4, 89.5, 110.8, 126.,9 128.7, 131.2, 133.6, 134.0, 143.0, 144.6,150.5,150.9,160.9,194.8.

**5-(3-hydroxyphenyl)-1,3,8,8-tetramethyl-5,8,9,10-tetrahydropyrimido[4,5-b]quinoline-2,4,6(1H,3H,7H)-trione**

White Solid; M.p: 303-305 °C;IR (KBr, cm^-1^): 3535, 3288, 3219, 3093, 2961, 1702, 1658, 1625, 1597, 1494, 1382, 1246, 783; ^1^H NMR (250 MHz, DMSO-*d_6_*): δ 0.87 (s, 3H), 1.00 (s, 3H), 2.01 (d, *J* = 15.75 Hz, 1H), 2.18 (d, *J* = 15.75 Hz, 1H), 2.49 (d, *J* = 11.75 Hz, 2H), 3.07 (s, 3H), 3.41 (s, 3H), 4.77 (s, 1H), 6.43 (d, *J* = 7.25 Hz, 1H), 6.62 (s, 2H), 6.88-6.94 (m, 1H), 8.96 (s, 1H), 9.08(s, 1H) ; ^13^C NMR (DMSO-*d_6_*, 62.5 MHz): δ 26.9, 28.0, 29.5, 30.06, 32.5, 33.8, 50.5, 90.5, 112.1, 113.2, 115.3, 118.6, 129.0, 144.1, 148.0, 149.8, 157.1, 161.1, 194.9.

**5-(3-hydroxyphenyl)-1,3,8,8-tetramethyl-5,8,9,10-tetrahydropyrimido[4,5-b]quinoline-2,4,6(1H,3H,7H)-trione**

White Solid; M.p: 303-305 °C;IR (KBr, cm^-1^): 3535, 3288, 3219, 3093, 2961, 1702, 1658, 1625, 1597, 1494, 1382, 1246, 783; ^1^H NMR (250 MHz, DMSO-*d_6_*): δ 0.87 (s, 3H), 1.00 (s, 3H), 2.01 (d, *J* = 15.75 Hz, 1H), 2.18 (d, *J* = 15.75 Hz, 1H), 2.49 (d, *J* = 11.75 Hz, 2H), 3.07 (s, 3H), 3.41 (s, 3H), 4.77 (s, 1H), 6.43 (d, *J* = 7.25 Hz, 1H), 6.62 (s, 2H), 6.88-6.94 (m, 1H), 8.96 (s, 1H), 9.08(s, 1H) ; ^13^C NMR (DMSO-*d_6_*, 62.5 MHz): δ 26.9, 28.0, 29.5, 30.06, 32.5, 33.8, 50.5, 90.5, 112.1, 113.2, 115.3, 118.6, 129.0, 144.1, 148.0, 149.8, 157.1, 161.1, 194.9.

**1,3,8,8-tetramethyl-5-(p-tolyl)-5,8,9,10-tetrahydropyrimido[4,5-b]quinoline-2,4,6(1H,3H,7H)-trione**

White Solid; M.p: 332-335 °C;IR (KBr, cm^-1^): 3281, 3220, 3090, 2961, 1702, 1662, 1603, 1507, 1380, 962, 756 ; ^1^H NMR (250 MHz, DMSO-*d*_6_): δ 0.86 (s, 3H), 1.01 (s, 3H), 1.99 (d, *J* = 16 Hz, 1H), 2.16 (s, 3H), 2.18 (d, *J* = 16 Hz, 1H), 2.50 (d, *J* = 12.5 Hz, 2H), 3.05 (s, 3H), 3.42 (s, 3H), 4.80 (s, 1H), 6.94 (d, *J* = 7.5 Hz, 1H), 7.07 (d, *J* = 7.5 Hz, 2H ), 8.94 (s, 1H) ; ^13^C-NMR (DMSO-*d*_6_, 62.5 MHz): δ 21.0, 26.9, 28.0, 29.5, 30.5, 32.5, 33.7, 50.5, 90.7, 112.2, 127.9, 128.6, 135.2, 143.9, 144.1, 149.7, 150.9, 161.1, 194.9.

**5-(4-methoxyphenyl)-1,3,8,8-tetramethyl-5,8,9,10-tetrahydropyrimido[4,5-b]quinoline-2,4,6(1H,3H,7H)-trione**

White Solid; M.p: 307-309°C; ^1^H NMR (250 MHz, DMSO-*d*_6_): δ 0.86 (s, 3H), 1.01 (s, 3H), 2.00 (d, *J* = 16.00 Hz, 1H), 2.18 (d, *J* = 16.00 Hz, 1H), 2.48-2.54 (m, 2H), 3.06 (s, 3H), 3.41 (s, 3H), 3.64 (s, 3H), 4.78 (s, 1H), 6.70 (d, *J* = 8.00 Hz, 1H), 7.09 (d, *J* = 7.75 Hz, 1H), 8.94(s, 1H) ; ^13^C-NMR (DMSO-*d*_6_, 62.5 MHz): δ 26.9, 28.0, 29.5, 30.6, 32.5, 33.3, 44.7, 50.5, 55.3, 90.8, 112.3, 113.5, 125.2, 129.0, 139.1, 144.0, 149.6, 151.0, 157.8, 161.1, 195.0, 202.2 .

**5-(2-hydroxy-3-methoxyphenyl)-1,3,8,8-tetramethyl-5,8,9,10-tetrahydropyrimido[4,5-b]quinoline-2,4,6(1H,3H,7H)-trione**

White Solid; M.p: 298-300°C; IR (KBr, cm^-1^): ; ^1^H NMR (250 MHz, DMSO-*d*_6_): δ 0.91 (s, 3H), 1.02 (s, 3H), 2.04 (d, *J* = 15.75 Hz, 1H), 2.23 (d, *J* = 15.75 Hz, 1H), 2.57 (d, *J* = 8.75 Hz, 2H), 3.08 (s, 3H), 3.67 (s, 3H), 4.96 (s, 1H), 6.55-6.64 (m, 3H), 9.09 (s, 1H), 9.15(s, 1H); ^13^C-NMR (DMSO-*d*_6_, 62.5 MHz): δ 27.0, 28.3, 29.4, 30.8, 32.5, 50.3, 55.9, 110.5, 119.6, 120.9, 134.3, 151.4, 196.0.


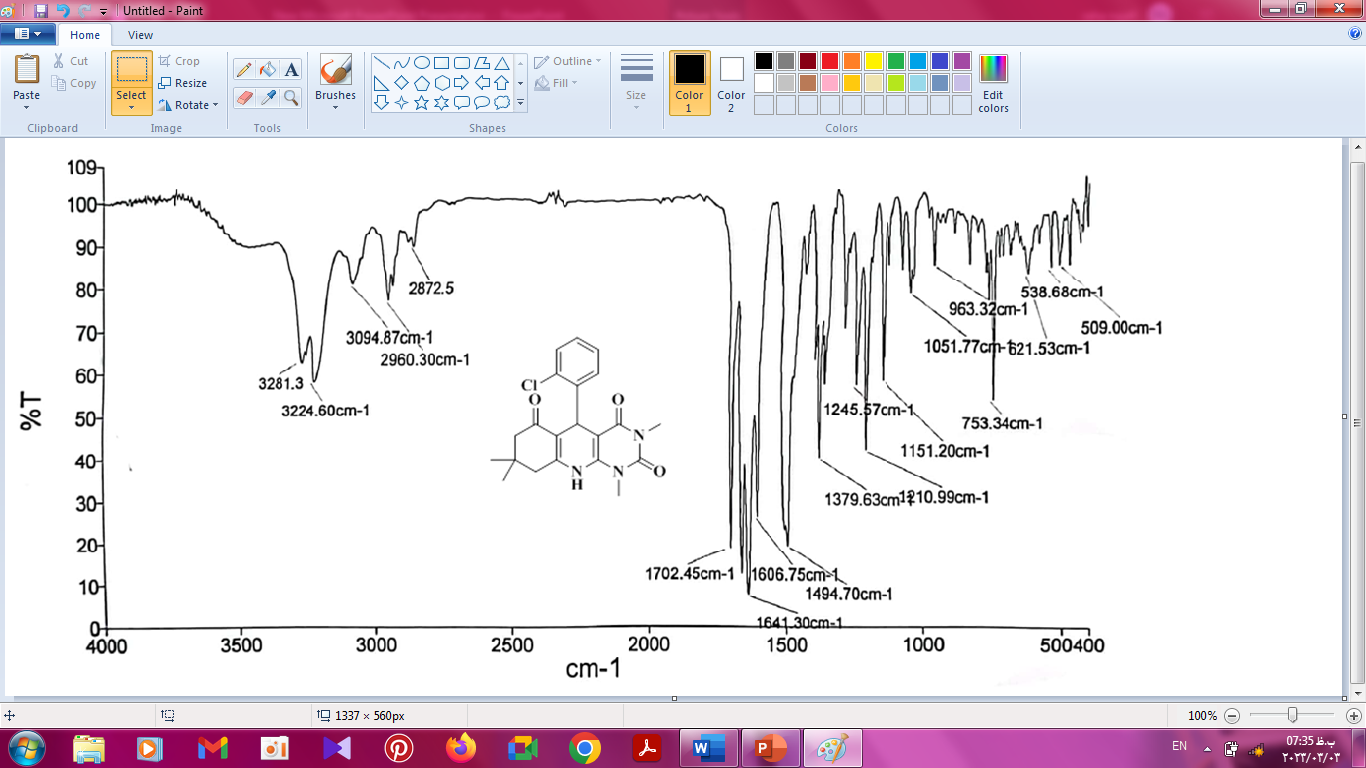


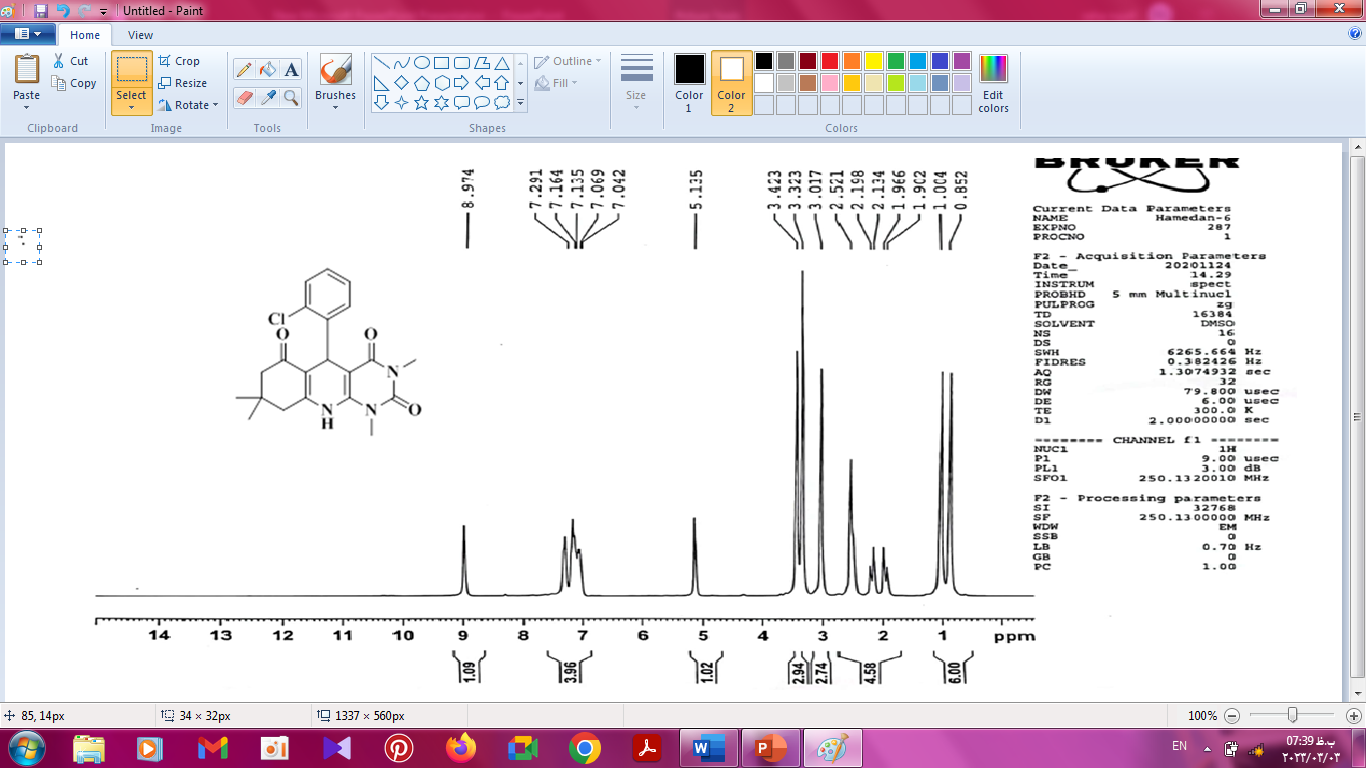


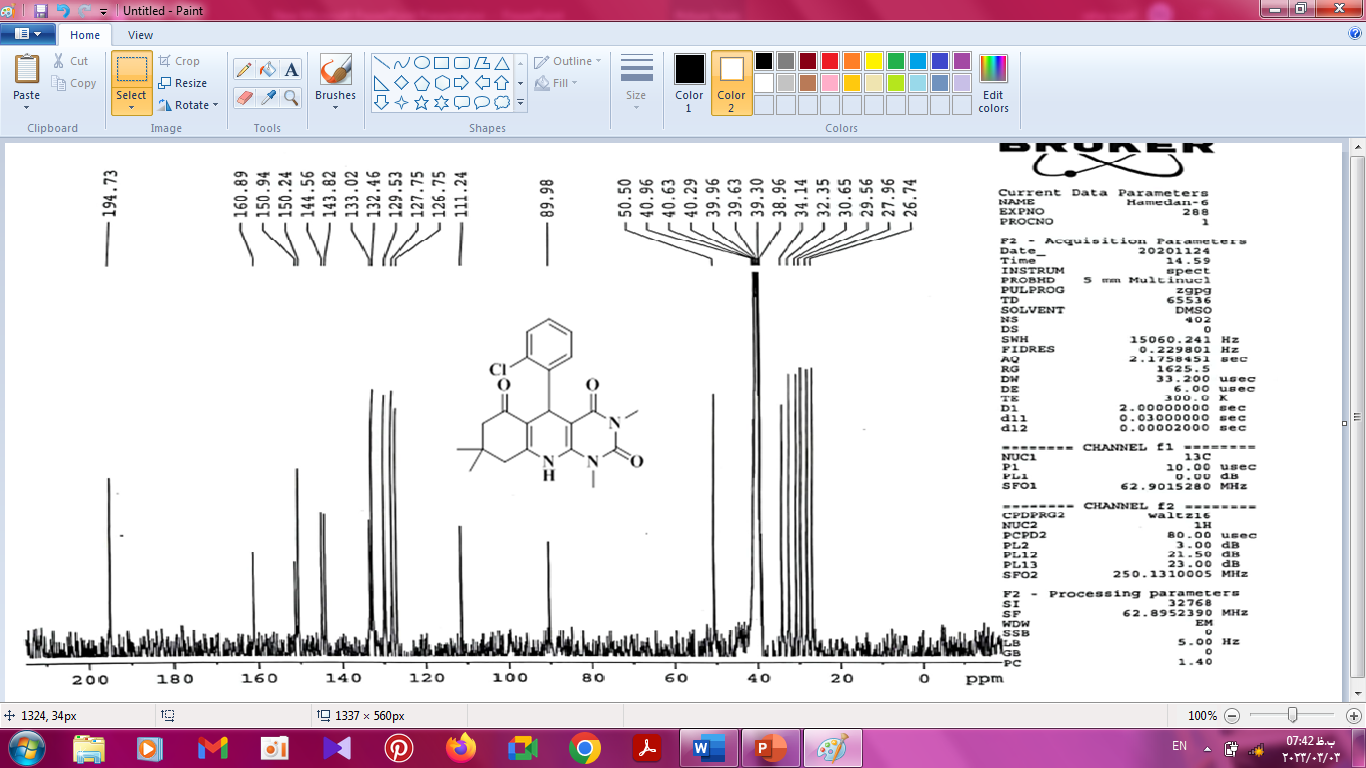


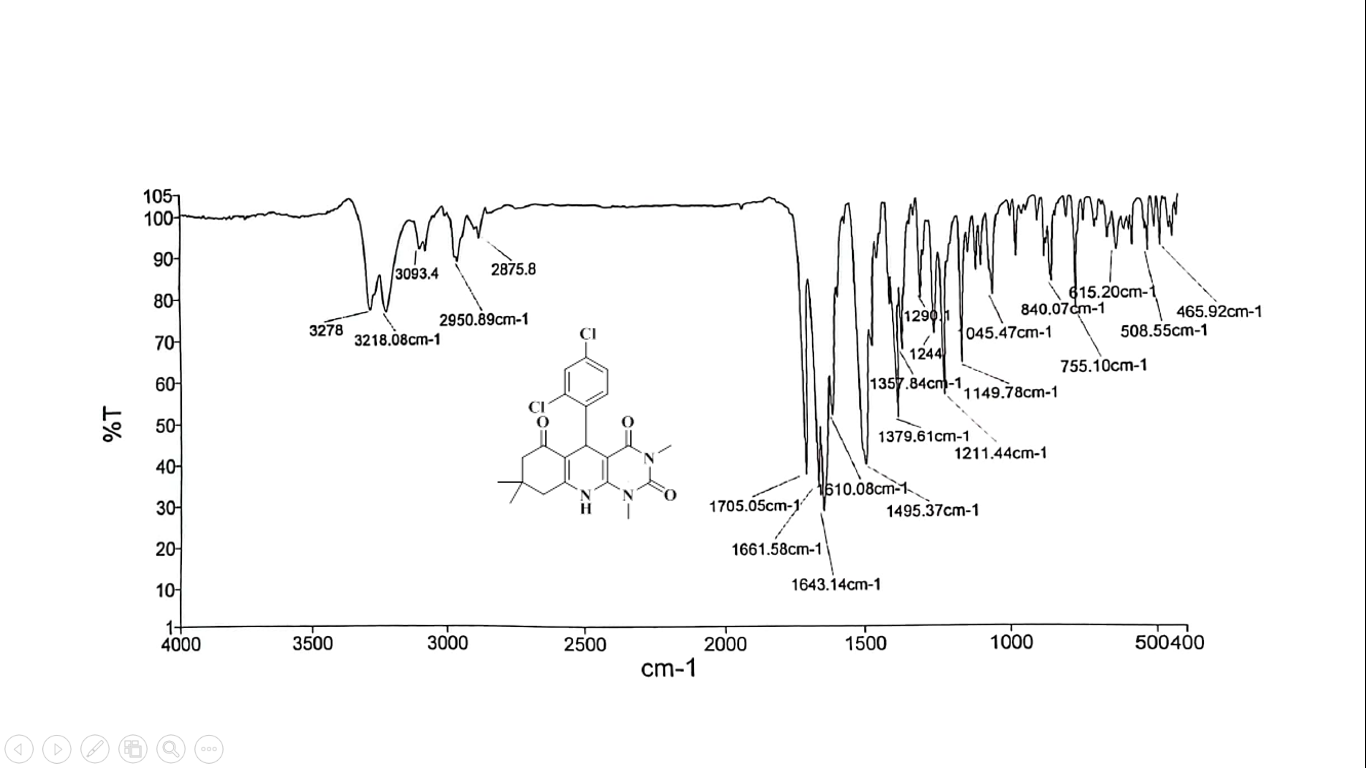


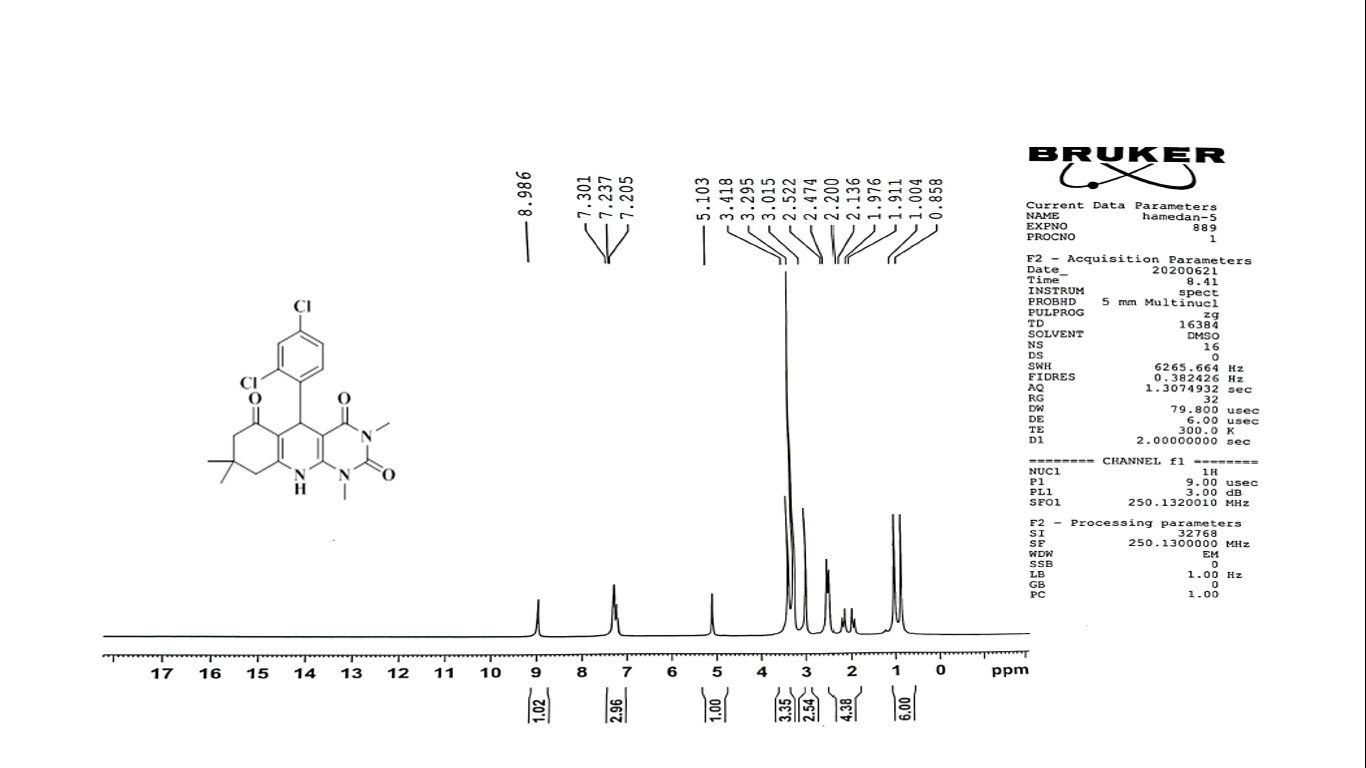


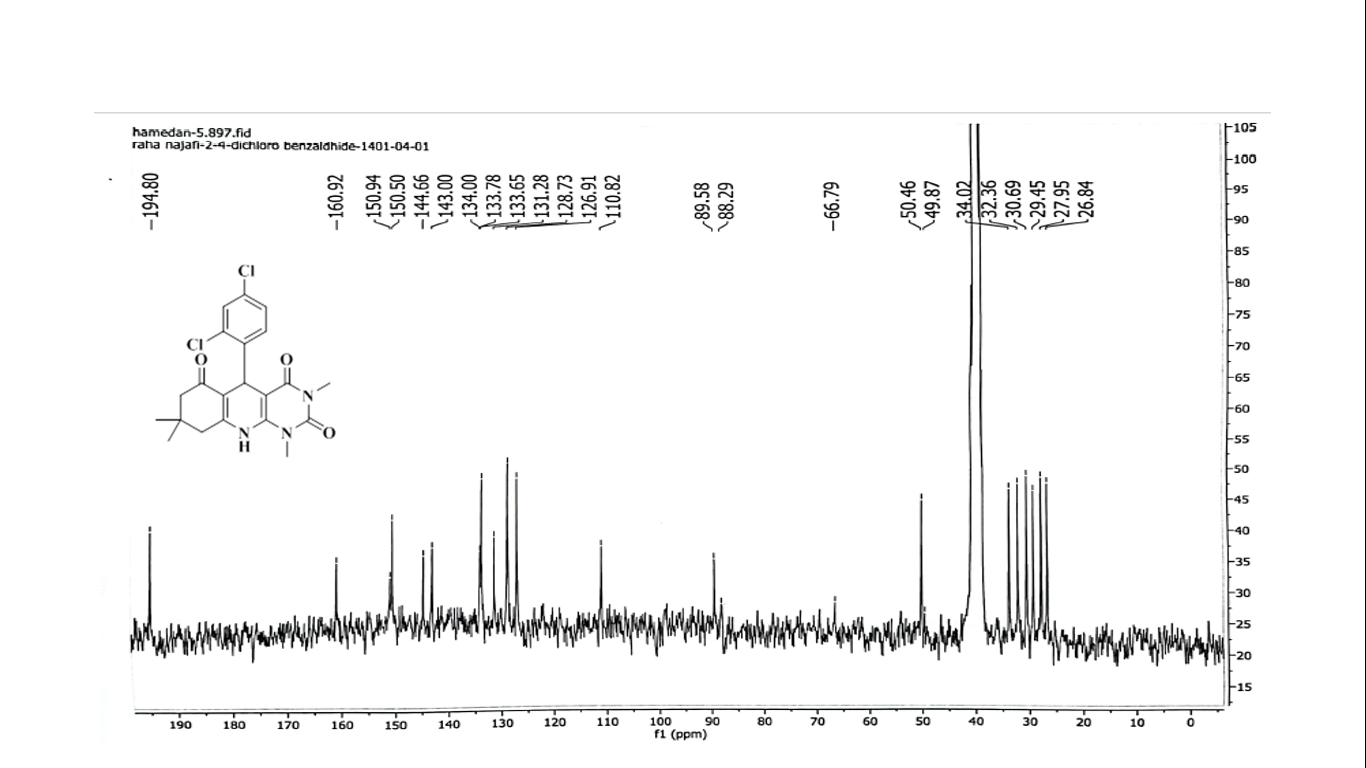


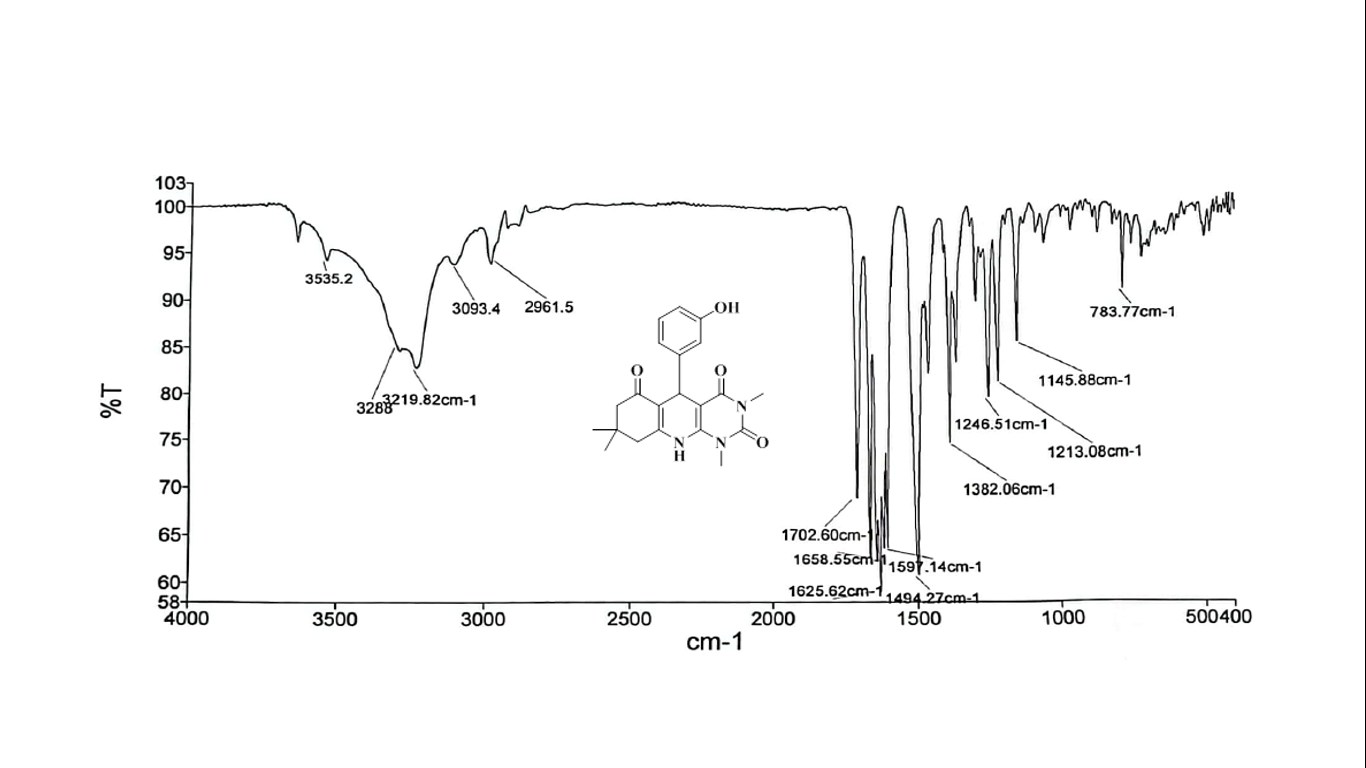


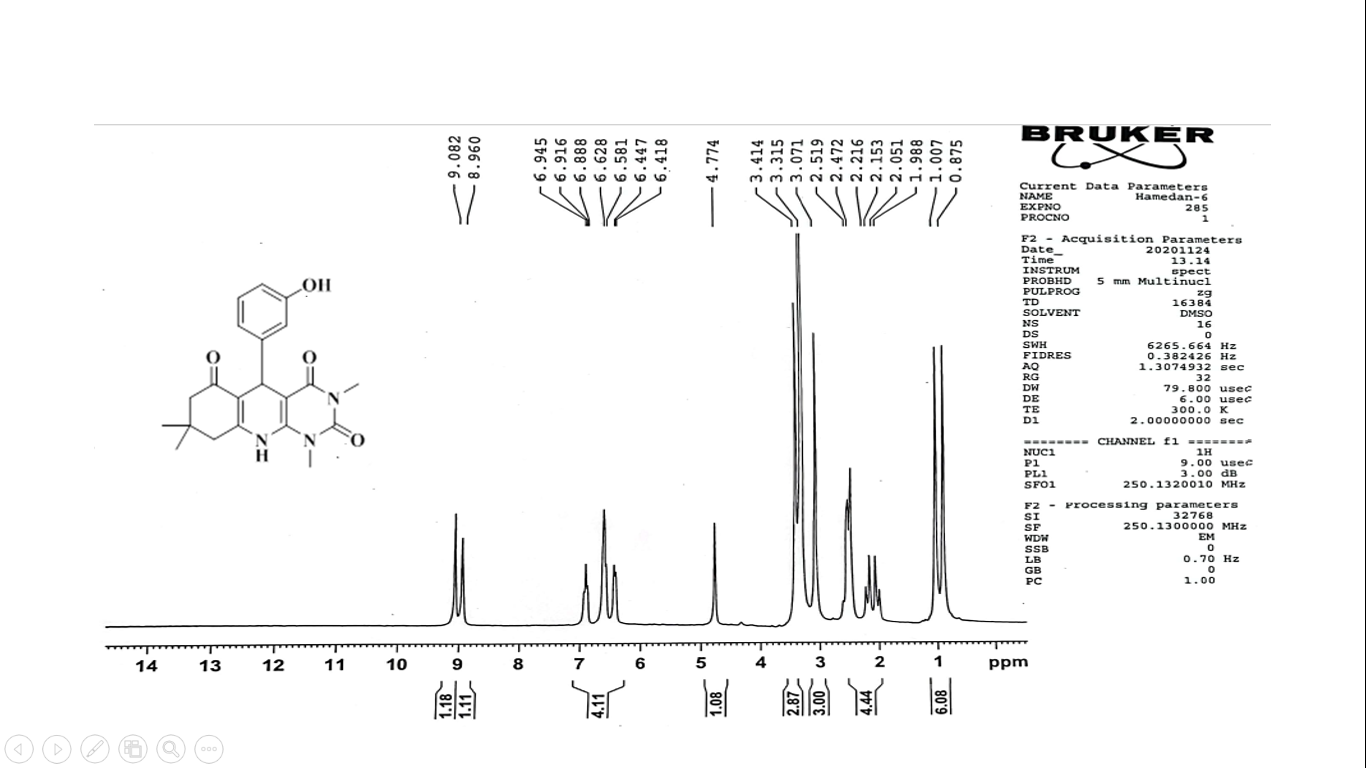


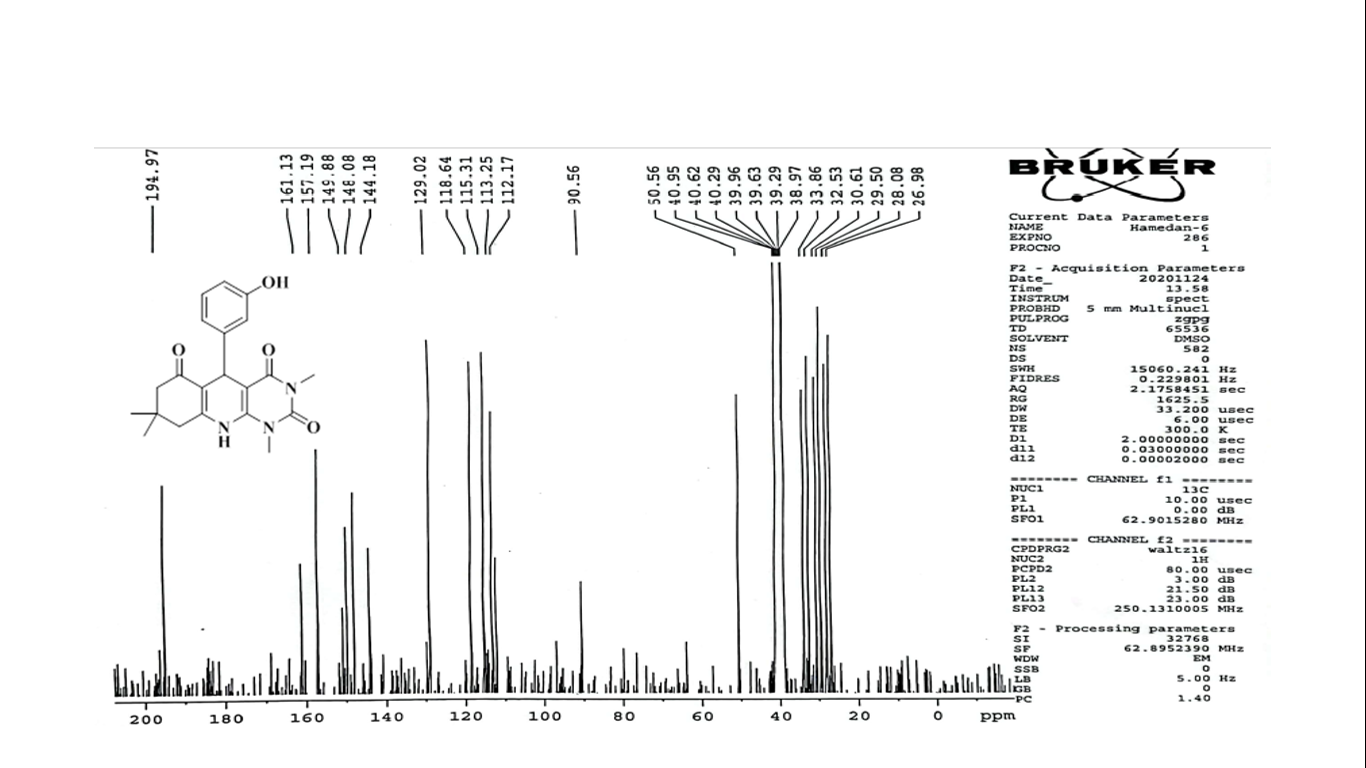


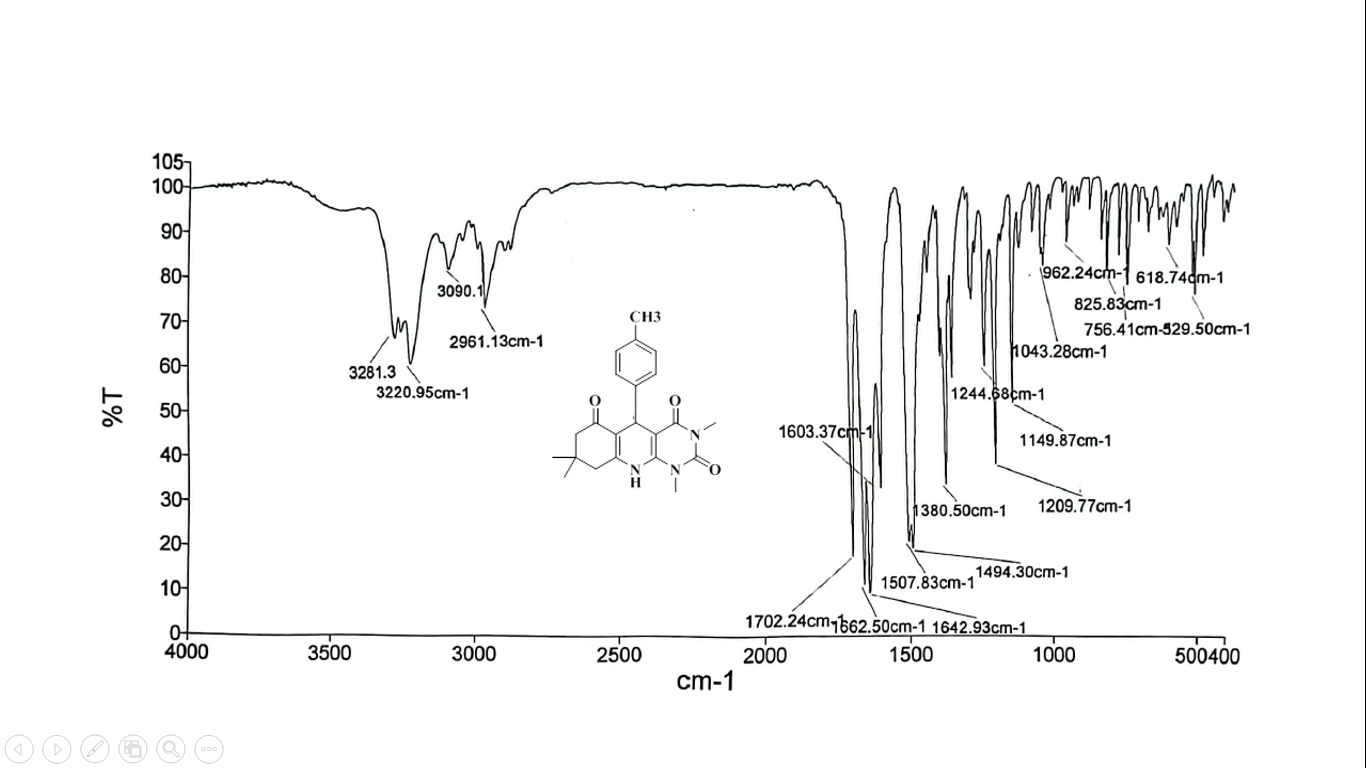


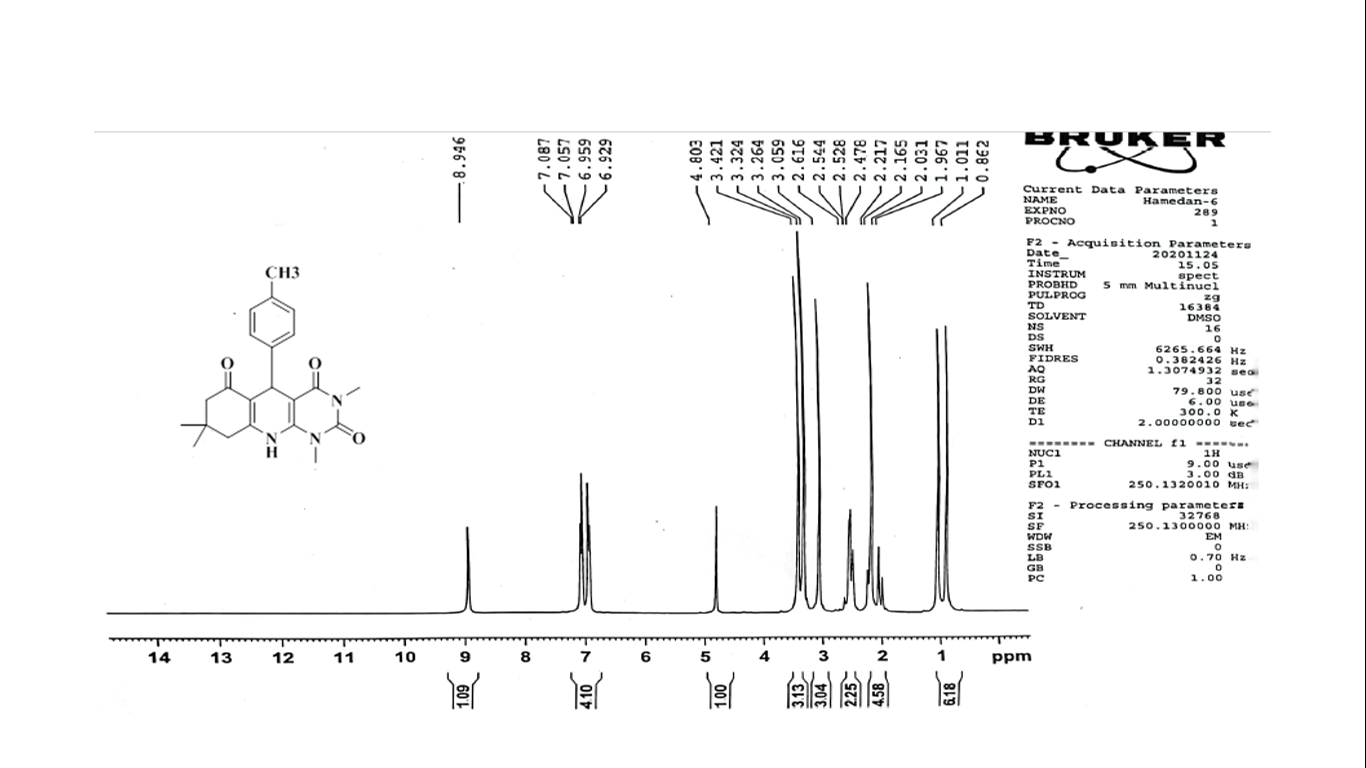


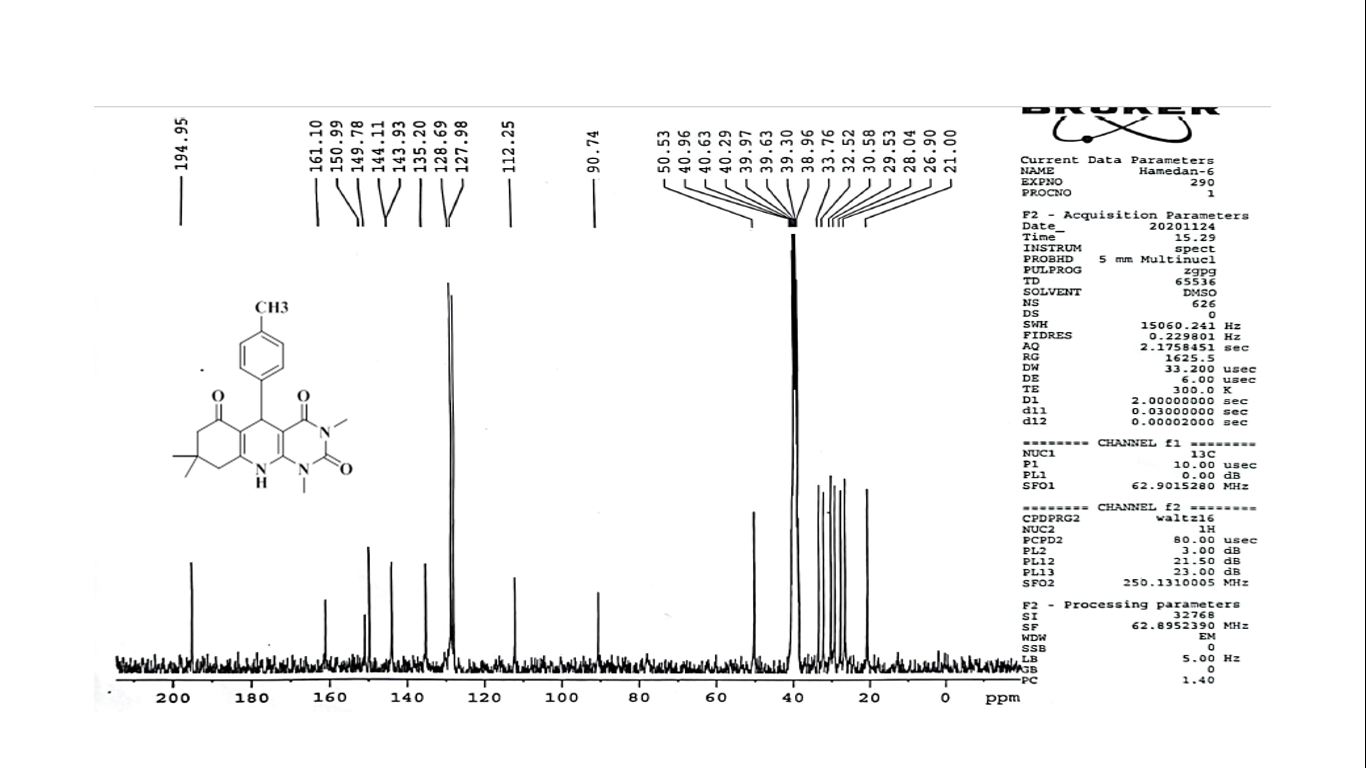


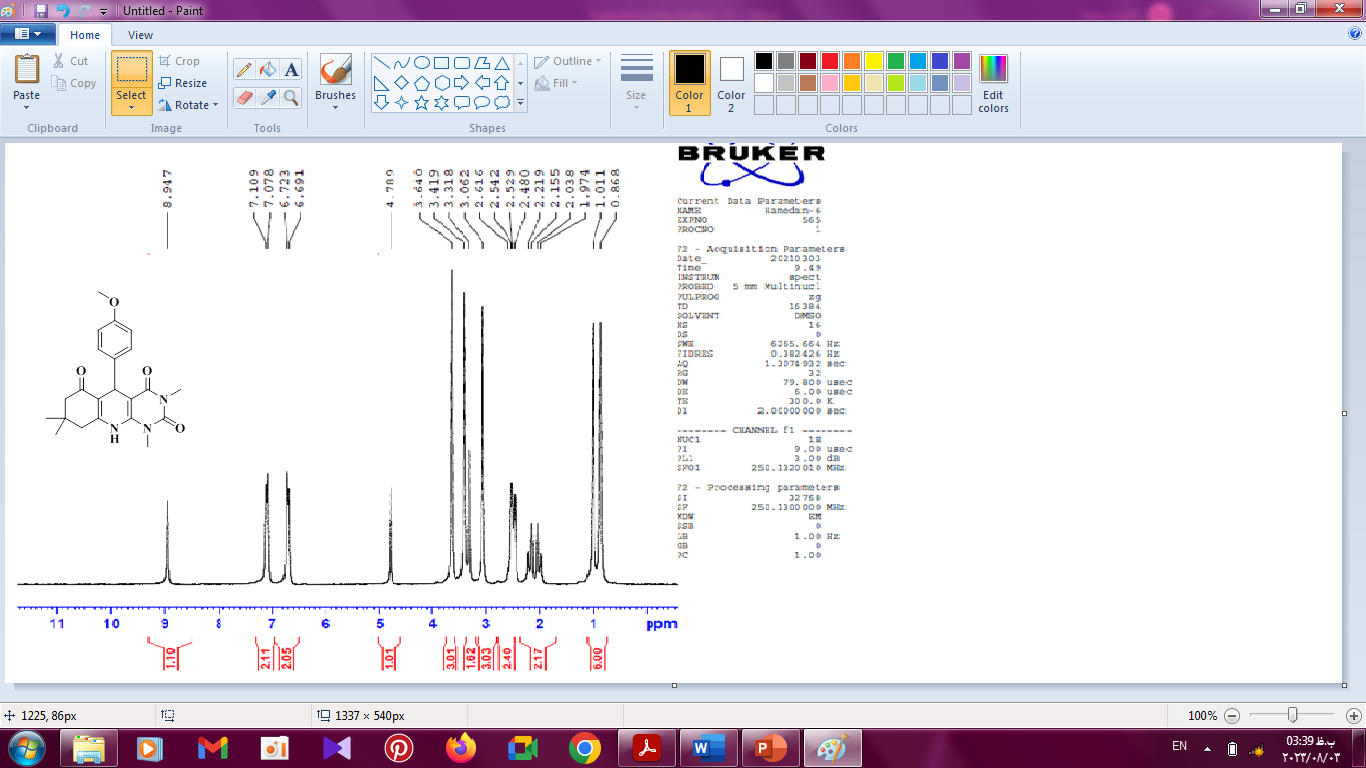


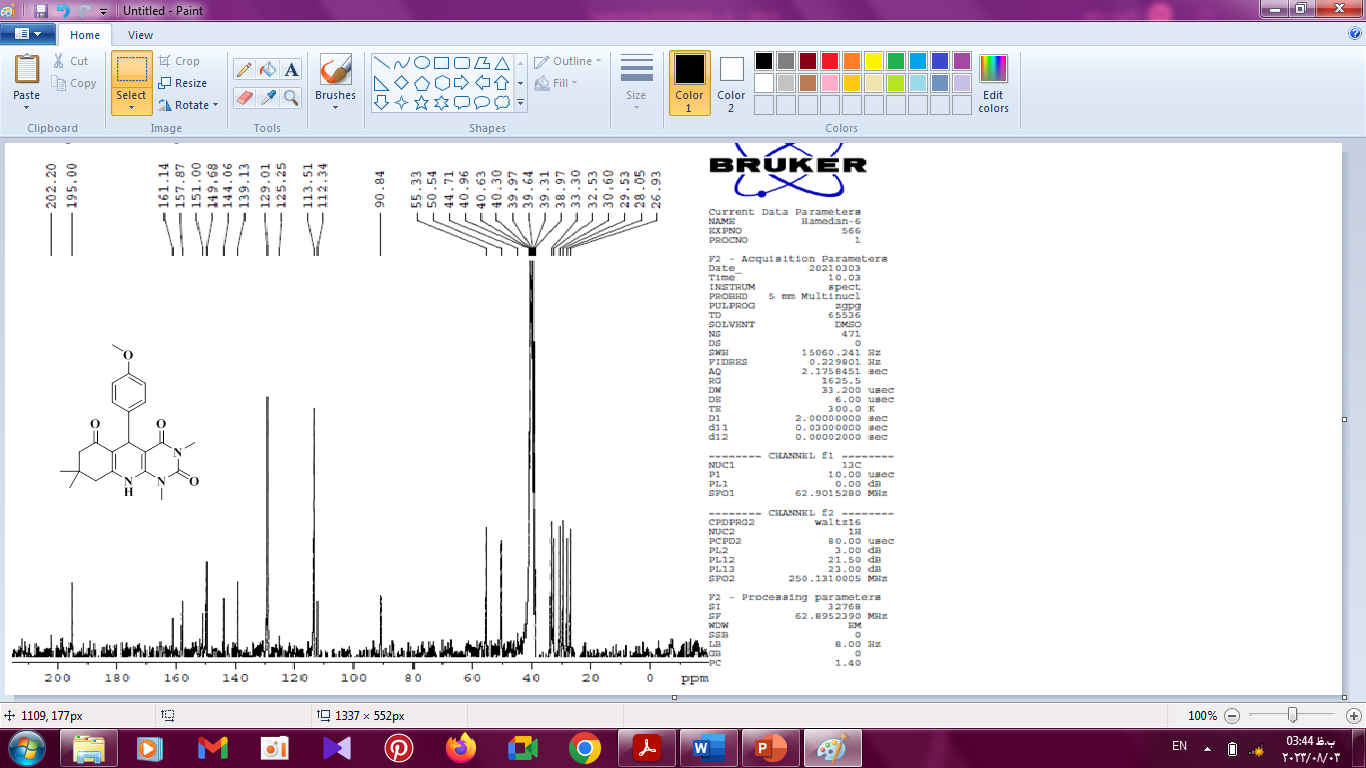


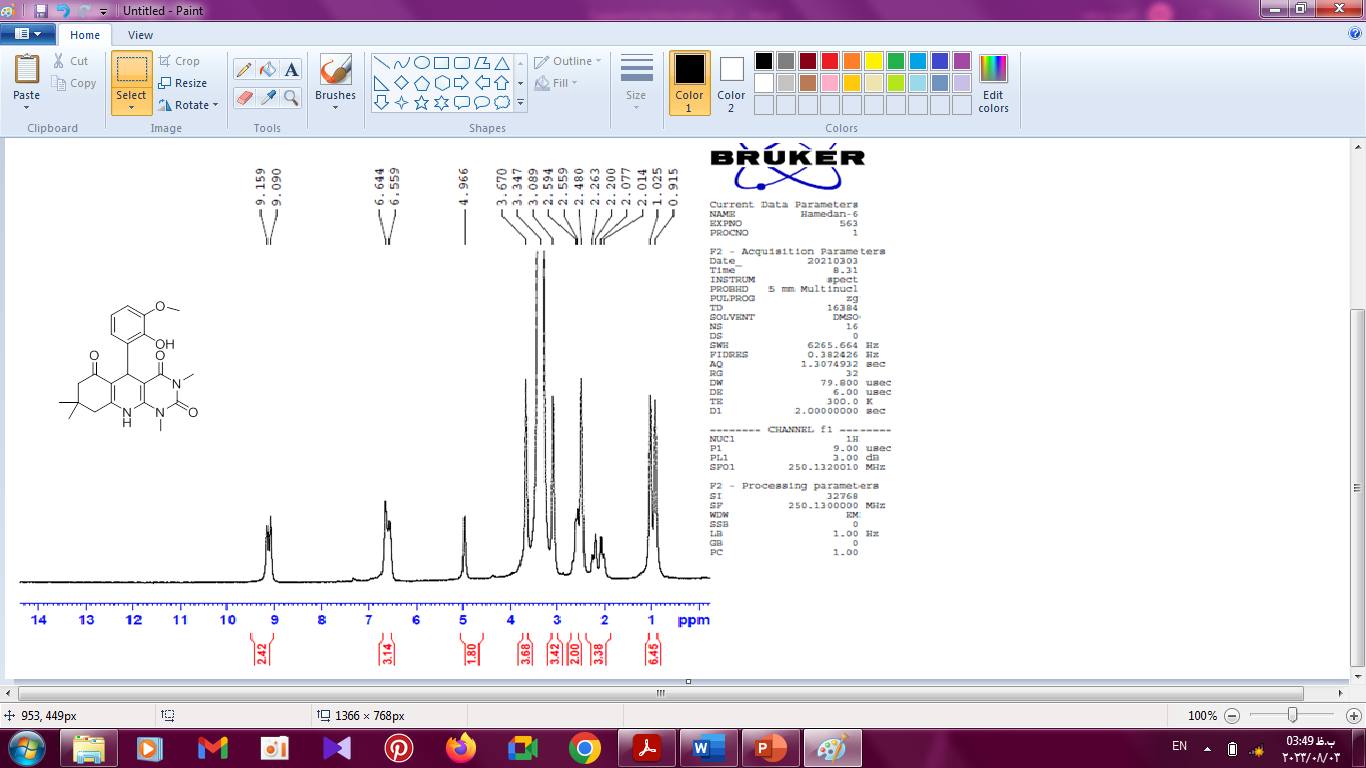


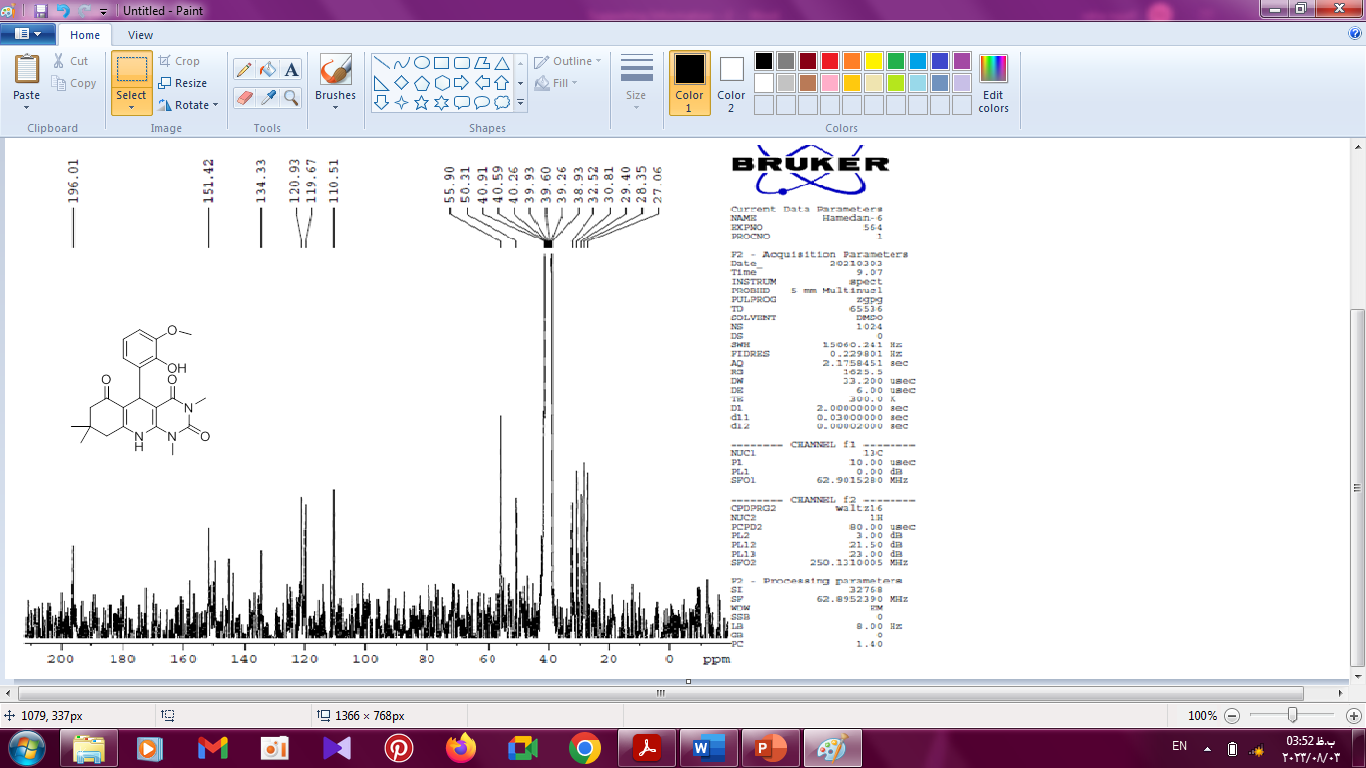

Supplement: Supplementary file 1 — Supplementary Information. [file 41598_2023_43793_MOESM1_ESM.docx]
